# A Natural Language Interface Concordant with a Knowledge Base

**DOI:** 10.1155/2016/9174683

**Published:** 2016-01-21

**Authors:** Yong-Jin Han, Seong-Bae Park, Se-Young Park

**Affiliations:** School of Computer Science and Engineering, Kyungpook National University, 80 Daehakro, Buk-gu, Daegu 41566, Republic of Korea

## Abstract

The discordance between expressions interpretable by a natural language interface (NLI) system and those answerable by a knowledge base is a critical problem in the field of NLIs. In order to solve this discordance problem, this paper proposes a method to translate natural language questions into formal queries that can be generated from a graph-based knowledge base. The proposed method considers a subgraph of a knowledge base as a formal query. Thus, all formal queries corresponding to a concept or a predicate in the knowledge base can be generated prior to query time and all possible natural language expressions corresponding to each formal query can also be collected in advance. A natural language expression has a one-to-one mapping with a formal query. Hence, a natural language question is translated into a formal query by matching the question with the most appropriate natural language expression. If the confidence of this matching is not sufficiently high the proposed method rejects the question and does not answer it. Multipredicate queries are processed by regarding them as a set of collected expressions. The experimental results show that the proposed method thoroughly handles answerable questions from the knowledge base and rejects unanswerable ones effectively.

## 1. Introduction

The studies in the field of natural language interfaces (NLIs) have attempted to match natural language questions with formal queries for easy access of information stored in a knowledge base. For example, let us consider the following query written in a functional query language [1]:

 
(answer (river (traverse_2 (state (loc_1(lowest (place (loc_2 (countryidusa:e))))))))).

A possible question that can be matched with this formal query is as follows: “Which rivers run through the state with the lowest elevation in the USA?” However, it is not easy for casual users to write such a question in a formal query language. Therefore, various types of query interfaces such as menu-guided interfaces [2], graphical query composers [3], and NLIs [4] have been proposed to improve the accessibility to knowledge bases. Among these query interfaces, NLIs allow more precise query expressions to be written with relative ease because users are more familiar with word-based query interfaces than with other graphical- or menu-driven interfaces. Hence, NLIs that translate natural language questions into their corresponding formal language queries have been studied extensively [5].

Many NLIs are based on syntactic parsing and semantic analysis for processing natural language questions [6]. However, current natural language understanding is not sufficient to process various kinds of natural language questions in practice. Even the state-of-the-art parsers produce many parsing errors that then propagate into semantic analysis and query generation, thus producing incorrect answers. The NLIs that are not based on natural language understanding also suffer from incorrect question processing. For example, classification-based methods depend on a classifier, while the classifier is usually not perfect [7].

**Figure 1:**
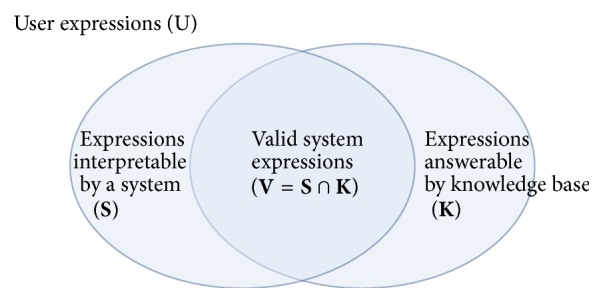
Mismatch between expressions that are interpretable by an NLI system and those that are answerable by a knowledge base.

The primary limitation of the current NLI systems is shown in Figure 1. In this figure, **S** is a set of expressions that are interpretable by an NLI system, and **K** is a set of expressions that can be answered by a knowledge base. The capability of the NLI system is defined as **V**, the intersection of **S** and **K**. There is a critical area (**K** − **S**) in which the system fails to interpret expressions but the knowledge base is indeed able to answer expressions. This area corresponds to the expressions that experience syntactic or semantic failure during their analysis. The area (**K** − **S**) can be reduced by expanding **S**. However, this requires highly accurate natural language processing techniques, which are, in general, impractical.

In order to resolve the discordance between **S** and **K**, this study proposes a novel method for NLIs. In the proposed method, instead of expanding **S** to cover as much of **K** as possible, **S** is generated from **K**. Thus, all the expressions in **S** can be answered because **S** is derived from **K**. The questions that are located outside **K** are rejected because they are also outside **S**. That is, the proposed method focuses only on user questions that are answerable by the knowledge base. In order to achieve this objective, each formal query is regarded as a query that can be generated from a graph-based knowledge base. That is, a formal query is represented as a graph having edges that are predicates defined in the knowledge base and nodes that are entities or concepts. Thus, all required formal queries can be generated as subgraphs of the knowledge base prior to query time, and then all possible natural language expressions corresponding to each formal query can also be collected in advance.

A natural language question has a one-to-one mapping with a formal query. Hence, a natural language question is translated into a formal query by matching the question with the most appropriate expression. If the confidence of this matching is not sufficiently high, the proposed method rejects the question and does not answer it. Multipredicate queries are processed by considering them as a set of collected expressions. Thus, the discordance is solved by focusing only on the expressions for concepts or predicates existing in the knowledge base.

The benefits of the proposed method are threefold. First, the proposed method can answer almost all questions obtainable from a knowledge base because the questions are systemically generated from the knowledge base. Second, unexpected complex questions are also processed. This can be easily accomplished by combining the prepared single predicate expressions. Third, unanswerable questions can be simply rejected when matching confidence is low.

The remainder of this paper is organized as follows.  Section 2 describes related work. In Section 3, the outline of the proposed method is described. The proposed method has two stages: (1) generation of system-interpretable expressions from a knowledge base and (2) translation of user questions into formal queries by using the generated expressions, explained in Sections 4 and 5, respectively. In Section 6, the experimental results of an NLI system built with the proposed method are discussed and compares the results from other existing NLI systems. Finally, the conclusion is presented in Section 7.

## 2. Related Work

### 2.1. Syntactic Parser-Based NLIs

Most studies on in the field of NLIs are based on natural language processing techniques. Early parsing-based systems used a syntactic parser to write rules for mapping from a natural language question to a formal query [6]. A rule is a simple mapping of a word in the question to a formal query component or a mapping of the syntactic structure of the question to the structure of the corresponding formal query. Therefore, the performance of syntax-based systems relies on the coverage and accuracy of mapping rules and the performance of the underlying syntactic parser.

In order to improve the performance of syntax-based systems, many studies were conducted to develop the mapping rules adapted to an ontology, a knowledge base [4, 8, 9]. In Orakel [4], the rules aim to map a syntactic structure to a semantic structure provided by an ontology and they are written manually. In order to simplify writing of the rules, Orakel provides a graphical user interface (GUI) to link an expression and its syntactic structure with an ontology predicate. The experiments in this study showed that an iterative customization of rules by the GUI steadily improves the performance of NLI.

AquaLog [8] and PANTO [9] also use a syntactic parser for translating a user question into a formal query. In these methods, a user question is translated by decomposing the question into linguistic triples. A syntactic parser is used to identify a linguistic triple that relates two terms or phrases with a verb or preposition. Then, each identified linguistic triple is mapped to an ontological triple. During this mapping, the terms in the linguistic triple are matched with the terms in the ontology. However, several discrepancies exist between linguistic triple terms and ontology terms. In order to solve this problem, AquaLog and PANTO learn new terms corresponding to ontological terms such as concepts or predicates by obtaining the mappings between linguistic and ontological terms from the user. A user question is translated into a final formal query by combining the identified ontological triples.

PRECISE [10] answers only semantically tractable questions for relational databases. The semantically tractable question is obtained from a lexicon and a set of strict constraints to define correct meaning composition. For example, a question is semantically tractable if it is converted to a unique sequence of tokens and the tokens are mapped to database elements (e.g., attributes or values) that are compatible. A question is tokenized by a syntactic parser and a lexicon that matches tokens with database elements is created manually in advance. Hence, PRECISE guarantees correct mapping of the question to its corresponding SQL query if the question is semantically tractable.

The syntactic parser-based NLIs generate a formal query from query elements which are obtained in advance by analyzing a user question with a syntactic parser. Unlike these syntactic parser-based NLIs, our proposed approach generates formal queries answerable by a knowledge base prior to query time and at least one expression corresponding to a formal query is generated in advance. As a result, a user question which matches a prepared expression exactly becomes semantically tractable one as in PRECISE. However, the proposed method can generate a formal query unlike PRECISE even when there is no expression matched to the question exactly. That is, a user question can be mapped to an appropriate formal query by the proposed method if a matching is confident enough.

### 2.2. Semantic Grammar-Based NLIs

Semantic grammar-based NLIs translate a user question into a formal query by parsing the question and mapping it into a formal query like the syntactic parser-based NLIs. The difference between semantic grammar- and syntactic parser-based NLIs is related to the nonterminal nodes of a parse tree. Semantic grammar-based NLIs use special semantic markers for nonterminal nodes instead of syntactic markers [6]. This allows a formal query to be generated directly from a parse tree instead of mapping rules.

Early semantic grammar NLIs such as Chat-80 [11] and LUNAR [12] built a hand-crafted grammar for a particular knowledge base; however, it is difficult to port such grammar to other knowledge bases. Hence, methods to learn semantic grammar automatically have been studied [13, 14]. These methods use training data consisting of a set of pairs of a natural language question and its formal query and learn a set of rules that map the terms and syntactic patterns to their formal query components. Given a sentence, the rules are applied to derive the most probable formal query. However, the creation of training data is difficult and time-consuming. Thus, response-driven learning methods [15, 16] have been proposed in order to reduce the supervision efforts. These methods utilize user feedback about the execution of the translated formal query producing the desired answer for the given question. Recent studies [17, 18] employed a paraphrase corpus to generate training data. The corpus used is constructed from the collaboratively edited QA site WikiAnswers, and, hence, questions only observed in the site are considered.

A common principle of semantic grammar-based NLIs is to learn grammatical rules from questions corresponding to formal queries. Though the proposed method also utilized user questions, its purpose is to match a user question to one of the prepared questions. As a result, the user question is mapped to a formal query corresponding to the matched question. This is achieved efficiently by regarding the questions which correspond to multipredicate formal queries as a set of simple expressions for concepts or predicates of a knowledge base. Thus, in the proposed method, the questions required are just simple expressions, whereas the questions corresponding to multipredicate formal queries must be prepared in semantic grammar-based NLIs.

### 2.3. Classification-Based NLIs

In NLIs based on a syntactic parser, parsing errors of a user question are propagated to semantic analysis and query generation, thus producing an incorrect answer. In order to overcome this limitation, the classification-based approach was proposed. The classification-based approach has been extensively studied for dialog systems [7, 19]. It trains classifiers for predefined formal query components; a classifier for a component determines whether a user question contains the meaning of the component. A component includes a representation regarding other components that can be combined with it, and hence formal query candidates are generated as possible combinations of the selected components. Each candidate is assigned a score by aggregating the classification confidences for its components. A final formal query is determined by ranking the candidates based on their scores.

QACID [20], the most well-known classification-based NLI system, uses a nearest neighbor classifier. It translates a user question into a formal query by determining the most appropriate cluster. A cluster is a group of semantically equivalent questions. Each cluster is associated with a formal query by a human annotator. Thus, a question is translated into a formal query by determining the most similar cluster. This cluster is found by using a nearest neighbor method.

The proposed approach belongs to a nearest neighbor classification because the semantics of a user question is determined by its most similar known expression. One of the key issues in classification-based NLIs is how to collect a number of good examples. The proposed approach for an NLI provides a systematic method to collect examples. We focus on natural language phenomena in the simple expressions that correspond to concepts or predicates of a knowledge base. That is, such simple expressions are collected, and then the semantics of an expression is determined by matching them with the most appropriate expression collected in advance. Unexpected complex expressions are effectively handled by combining the collected expressions.

## 3. Overall Structure

**Figure 2:**
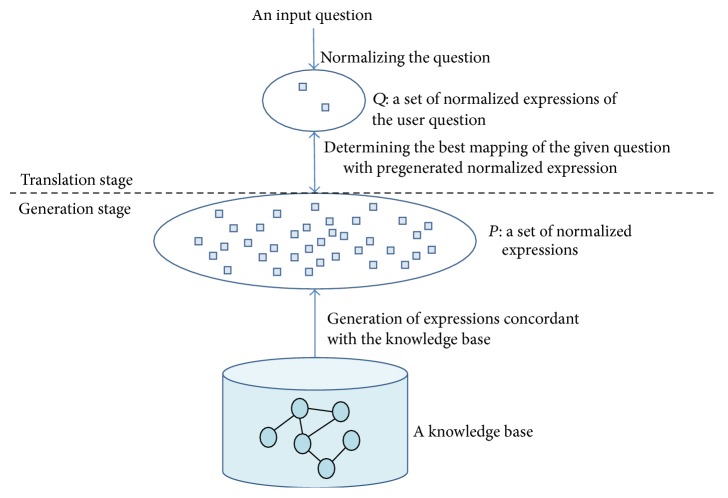
An overview of our proposed approach.

In this study, an ontology is used as a knowledge base; an ontology is an explicit specification of conceptualization and defines describable facts [21]. This specification is used as constraints when translating a user question into a formal query because the question must be concordant with the ontology to be answered [22]. An overview of our approach is conceptually illustrated in Figure 2. The approach consists of two stages: (1) the generation stage that generates expressions concordant with a knowledge base, and (2) the translation stage that translates user questions into formal queries by determining the best mapping of the user questions with the pregenerated expressions.

The generation stage prepares all possible natural language expressions that will be matched with a user question. These natural language expressions are generated from an ontology. An ontology has a graph structure with vertices and edges labeled in natural language. Thus, all substructures in the ontology can be expressed in natural language. We generate at least one natural language expression for each subgraph of the ontology. The expressions are generated using the ontology schema, not using ontology instances, because the schema represents all describable meanings. Every expression is converted into a token sequence to be matched with a user question and the sequence is called as a* normalized expression*.

In the translation stage, a user question is translated into a formal query by determining the normalized expression that is semantically equivalent to the question. First, a user question is converted into several normalized expressions owing to its various ambiguities. For example, an entity, Mecklenburg, can be recognized as two concepts,* region* and* castle*, when the knowledge base archives it as a region and a castle. Thus, a question can be transformed into one or more normalized expressions. Next, the meaning of the question is determined by selecting the most appropriate pair of normalized expressions from the question and the knowledge base. A final formal query is formulated using the selected pair and the subgraph of the knowledge base that corresponds to the pair. When *P* is a set of normalized expressions that are generated from the knowledge base and *Q* is a set of normalized expressions from the user question, the correct pair (*p* ^*∗*^, *q* ^*∗*^) among all possible pairs from *P* and *Q* is determined by

(1) $$\begin{array}{c} \left(\;p^{*},q^{*}\;\right)=\underset{\left(p,q\right)\in P\times Q}{arg\;max\;}\text{Sim}\left(\;p,q\;\right), \end{array}$$

where Sim(*p*, *q*) is the similarity between *p* ∈ *P* and *q* ∈ *Q*.

## 4. Generating Expressions Answerable by a Knowledge Base

### 4.1. Subgraph Generation from Knowledge Base Schema

An ontology used as a knowledge base can be interpreted as a graph [1]. Since the schema represents all the meanings of an ontology, hence, a schema graph is actually used as a knowledge base. This schema graph is a labeled graph with labeled vertices and labeled edges. Consider *P* to be a set of all the subgraphs obtainable from a schema graph, *G* = (*V*, *E*), where *V* is a set of vertices and *E* is a set of edges. Then, the size of *P* is bounded as

(2) $$\begin{array}{c} \left|\;P\;\right|\leq \overset{n}{\underset{k=1}{\sum }}\left(\begin{array}{c} n\\ k \end{array}\right)+m, \end{array}$$

where *n* = |*E*| and *m* = |*V*| because any possible combination of the edges in *G* forms a subgraph if they are connected by one or more vertices. |*P*| increases exponentially as the number of edges increases. Thus, it is impractical to process all the subgraphs. Although certain information can be accessed using more than ten predicates, such information is extremely rare. Most user questions are typically short and use only a few predicates. According to the usability study of NLI [23], casual users use an average of three simple questions to access specific information. Therefore, in this paper, the maximum number of edges in the subgraph is restricted to three. Then, the number of possible subgraphs is bounded as

(3) $$\begin{array}{c} \left|\;P\;\right|\leq \overset{3}{\underset{k=1}{\sum }}\left(\begin{array}{c} n\\ k \end{array}\right)+m. \end{array}$$

The constrained generation of subgraphs is described in Algorithm 1. The algorithm returns** S**, a set of all possible subgraphs of the schema graph *G*_sch_ by generating subgraphs recursively. First, the algorithm generates a simple subgraph for each vertex in the schema graph in Line (4). Then, for all simple subgraphs, it generates more complex subgraphs with more edges by executing the function GenSubgraph.

In the function, GenSubgraph, if the number of edges is greater than three, the recursion terminates at Line (15) because the maximum number of edges is restricted to three; else, a new subgraph is generated by adding an edge to a pregenerated subgraph. This new subgraph is generally meaningful; however, sometimes, it may be invalid. For example, let us consider a subgraph with two isCityOf's. The predicate isCityOf connects two concepts, City and State, and the semantics of this predicate indicates that City is one of the cities in State. When these two isCityOf's are connected by City, the subgraph has no meaning because City belongs to two different states. However, if they are connected by State, the subgraph is meaningful because the semantics of this subgraph indicate that two cities belong to State.

In order to eliminate such invalid or redundant subgraphs, some* characteristics* of predicates are used as constraints in the generation of subgraphs. The constraints used are as follows:

(1) If two predicates have an inverse relationship, only one of them is used in subgraph generation.
(2) The predicate *p*(*a*, *b*) cannot be connected with *p*(*a*, *c*), if *p* is functional.
(3) The predicate *p*(*a*, *b*) cannot be connected with *p*(*c*, *b*), if *p* is inverse functional.

### 4.2. Generation of Expressions from Schema Subgraphs

A natural language question is a sentence, and hence it cannot be matched directly with schema subgraphs. In order to permit this matching, natural language expressions for each subgraph are prepared in advance. The expressions are created in two steps.

The first step is an automated one that collects at least one natural language expression for a simple concept or predicate. A natural language expression may not be a grammatically correct sentence. In order to enable easy matching of the natural language expressions with a user question, the expressions are normalized. For example, the normalized expression for a predicate flowThrough, labeled as “flows through,” that connects the concepts River and City is

 
〈River〉 flow through  〈City〉.

The normalized expression is only a sequence of tokens. The tokens are of two types: a lexical string and a semantic code. The lexical string of a word is the base form of this word and it is obtained by lemmatization. In the example above, “flow” and “through” are lexical strings. A semantic code corresponds to a vertex of a schema subgraph. It expresses a concept or a data type. The semantic code  〈River〉 in the example above expresses a concept, “river.”

A new normalized expression is added if a predicate has an inverse relation with another predicate. For example, assume that a predicate flowThrough has an inverse relation with a predicate hasRiver whose label is “has river.” The label of hasRiver is lemmatized as “have river.” Then, “river” in the lemmatized form is removed, because it is redundant with the semantic code,  〈River〉. Thus, a new normalized expression

 
〈City〉 have  〈River〉

is generated. If a predicate does not have a label, its identifier can be used instead of the label. In general, identifiers in an ontology are represented by a common pattern. For example, the predicate, hasRiver, originates from two terms, “has” and “river,” and the term “river” that follows the first term begins with a capital letter. Thus, hasRiver can be tokenized by inserting a space before each capital letter. Thus, a normalized expression can be generated from the tokenized identifier. The normalization process can be summarized in four steps as follows:

1. tokenization of an identifier;
2. lemmatization of an expression for the identifier;
3. elimination redundant terms;
4. assignment of a semantic code to the expression.

The expressions for a subgraph with multiple edges are generated by combining the expressions of the edges. Let *G* be a subgraph with *k* edges and let *e*_*i*_ be the *i*th edge of *G*. When *P*_*e*_*i*__ is the set of natural language expressions for *e*_*i*_, the set of natural language expressions for *G*, denoted as *P*_*G*_, is

(4) $$\begin{array}{c} P_{G}=P_{e_{1}}\times P_{e_{2}}\times \cdots \times P_{e_{k}}. \end{array}$$

**Figure 3:**
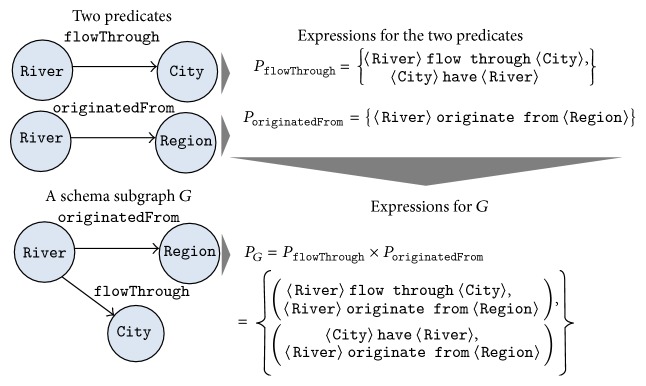
Generation of expressions for a subgraph with multiple edges.

For example, Figure 3 shows the manner in which the expressions from a subgraph with two edges are generated. The subgraph *G* in this figure has two predicates, flowThrough and originatedFrom. The set of the natural language expressions for flowThrough is  {〈River〉 flow through  〈City〉,  〈City〉 have  〈River〉}, while that for originatedFrom is  {〈River〉 originate from  〈Region〉}. Then, the expressions for *G* are obtained from their Cartesian product. Thus, *P*_*G*_, the set of expressions for *G*, is  {(〈River〉 flow through  〈City〉,  〈River〉 originate from  〈Region〉),  (〈City〉 have  〈River〉,  〈River〉 originate from  〈Region〉)}.

**Figure 4:**
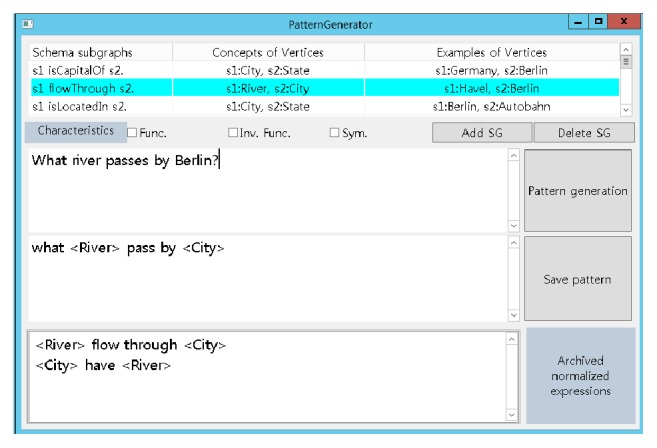
A screenshot of *PatternGenerator* for generation of new normalized expressions for a schema subgraph.

The second step aims to add various expressions manually to the automatically generated expressions. The expressions that are generated automatically are lexically limited, because they are based on the labels and identifiers. For example, let us consider the predicate flowThrough. A user question such as “what river flows through Berlin?” or “what river does Berlin have?” can be matched with  $P_{flowThrough}$, because there exists a similar expression. However, the question “what river passes by Berlin?” cannot be matched with  $P_{flowThrough}$, although the question is semantically the same as the elements of  $P_{flowThrough}$. In order to address this problem, additional expressions for each predicate are collected manually using an assistance program, *PatternGenerator*.  Figure 4 shows the corresponding screenshot. *PatternGenerator* lists all the schema subgraphs at the top. When the user provides a natural language question (in this figure, “what river passes by Berlin?”) for a subgraph, *PatternGenerator* lists all current expressions at the bottom. Further, it generates the normalized expressions for the question and displays them at the centre (in this figure, “what  〈River〉 pass by  〈City〉”). The user can save the generated expression if it is different from the current expressions. When multiple expressions are created owing to the ambiguity of the user question, *PatternGenerator* prompts the user to select the correct expression and saves the selected one.

Although a large number of schema subgraphs and their expressions are generated from an ontology, there exist some expressions that cannot be generated directly from the ontology but are answerable by the ontology. For example, let us consider a simple ontology that describes only a river and its length. Then, the question, “how many rivers are longer than Havel?”, can be answered by comparing the length of Havel with that of the other rivers. However, such an expression cannot be generated by the process explained above.

**Figure 5:**
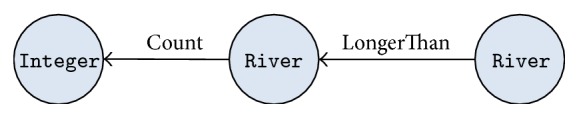
An example of a schema subgraph with metapredicates.

We solve this problem by allowing metapredicates proposed by Kate et al. [1]. For example, the question above can be answered with the two metapredicates, longerThan (River, River) and count (River), where The former represents a river being longer than another one, while the latter denotes the cardinality of a set of rivers.  Figure 5 shows the schema subgraph that answers the question above. A new schema subgraph such as this one and its normalized expressions can be created and collected manually with *PatternGenerator*. *PatternGenerator* has two buttons, “Add SG” and “Delete SG,” as shown in Figure 4. The “Add SG” button allows the user to write a new schema subgraph and the “Delete SG” button removes the current subgraph. Once a new subgraph is written, its normalized expressions are added by the user. Since schema subgraphs including metapredicates deliver their own meaning intuitively, even ordinary people can use *PatternGenerator* easily.

## 5. Translating a User Question into a Formal Query

### 5.1. Translation Process

The natural language question of the user is translated into a formal query by matching it with the most appropriate schema subgraph. The matching is actually performed by comparing the user question with the expressions prepared from the schema subgraphs. This translation process is represented in Algorithm 2. The algorithm begins with the normalization of a given user question in Line (4). The process of normalization is performed in the same manner as that in *PatternGenerator*. Owing to the ambiguities within the question, this step results in a set of normalized questions, *Q*.

**Figure 6:**
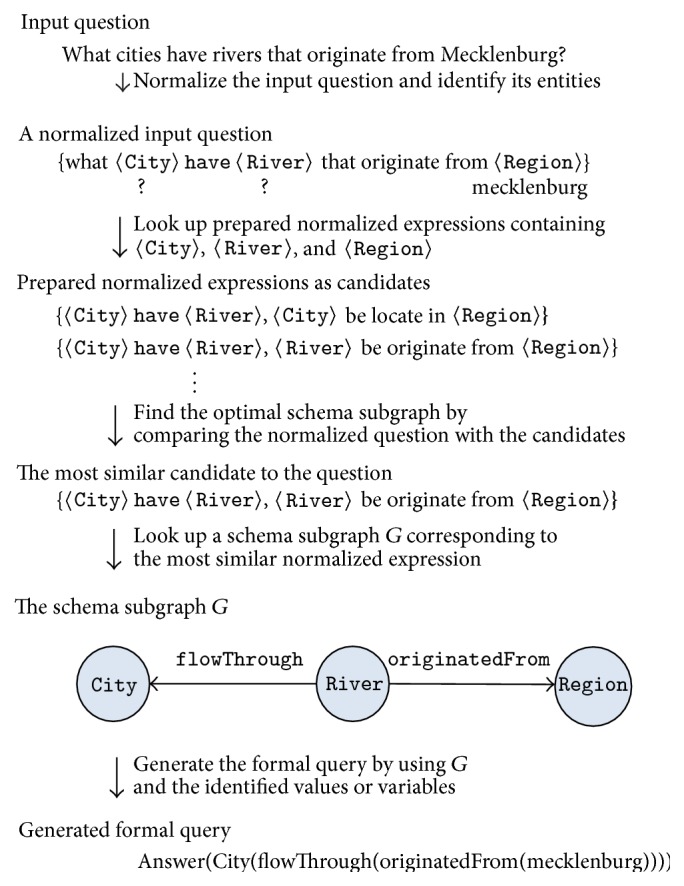
An example of translating a user question into a formal query.

Each normalized question *q* ∈ *Q* must be compared with all prepared normalized expressions. However, this is not computationally feasible. Therefore, the algorithm uses a simple heuristic method instead of examining all the prepared expressions. Only those expressions that share all the semantic codes of *q* are selected, and they form a set of candidates, *P*_*q*_. Then, for all *p* ∈ *P*_*q*_, the similarity between *p* and *q* is computed in Line (9). In Line (13), after sorting all (*p*, *q*, *s*)'s, the pair (*p* ^*∗*^, *q* ^*∗*^) with the greatest similarity is selected. If the greatest similarity is less than a predefined threshold *θ*, this algorithm rejects the user question. Else, a formal query is generated from the schema subgraph *g* that corresponds to *p* ^*∗*^. A functional query language is used as a formal language in this study, and hence the final schema subgraph can be easily converted into a formal query. We adopt the template-based method proposed by Zettlemoyer and Collins [24] to translate a schema subgraph into a formal query.  Figure 6 shows the entire translation process for the question “what cities have rivers that originate from Mecklenburg?”

### 5.2. Measurement of Expression Similarity

The primary step in the matching process is the measurement of the similarity between a normalized user question *q* and a prepared normalized expression *p*. We propose a subsequence kernel as a reliable measure of similarity. Subsequence kernel [25] is a well-known method for the comparison of string sequences or word sequences. It generates the similarity between two token sequences by matching all possible subsequences of the token sequences. Matching between two simple expressions can be performed with legacy subsequence kernels. However, a subgraph with multiple edges corresponds to a bundle of simple expressions. Thus, the similarity between *p* and *q* is computed for all simple expressions of *p*. Let *p* = (*p*_1_,…, *p*_*n*_) ∈ *P* be one of the prepared expressions and let *q* ∈ *Q* be a possible expression of a user question *u*. *p*_*i*_ ∈ *P* is a simple expression with a single predicate. Thus, when *p* has multiple predicates, that is, *n* > 1, the expression kernel between *p* and *q*, *K*(*p*, *q*), is defined as

(5) $$\begin{array}{c} K\left(\;p,q\;\right)=\overset{n}{\underset{i=1}{\sum }}k\left(\;p_{i},q\;\right), \end{array}$$

where *k* is a weighted string kernel proposed by Saunders et al. [26].

A basic assumption of the weighted string kernel is that tokens have varying significance. Thus, the decay factor for noncontiguous subsequence matching is weighted according to the type of token involved. In the expression kernel, we distinguish tokens into two types: a semantic code and a lexical term.

The time complexity of a string kernel is *O*(*l* | *q*|) where *l* is the average length of *p*_*i*_'s, and hence the time complexity of an expression kernel is only *O*(*nl* | *q*|), which is not computationally expensive.

*K*(*p*, *q*) is not bounded, and hence we use its normalized version for Sim(*p*, *q*) in Algorithm 2. Two methods are used for normalization of the kernel: (1) similarity Sim_NI_(*p*, *q*) is the normalization by inner product and (2) similarity Sim_EU_(*p*, *q*) is the inverse of the Euclidean distance. That is,

(6) SimNIp,q=Kp,qKp,pKq,q,

(7) SimEUp,q=1Kp,p+Kq,q−2Kp,q.

## 6. Experiments

The experiments aim to examine the effectiveness of the proposed method in reducing the discordance between the questions interpretable by a system and those answerable by a knowledge base.

### 6.1. Data and Evaluation

The US geography dataset [27] is used to evaluate the proposed method. It includes a geography knowledge base in Prolog and GeoQuery dataset. The GeoQuery dataset consists of pairs of a natural language question and its corresponding formal query. The proposed method uses the ontology as a knowledge base, and hence the ontology version of the Prolog knowledge base is used. The geography knowledge base in Prolog was translated into an OWL ontology by the DDIS research project [5]. The geography ontology has eight concepts, 17 relation predicates, and 11 datatype predicates. Eight relation predicates among the 17 predicates have an inverse relation. For example, hasRiver has an inverse relation with runsThrough. The remaining predicate is borders, a symmetric predicate. The GeoQuery dataset has two versions. One is Geo880, a full GeoQuery dataset with 880 pairs of natural language and formal queries. The average length of the question and the formal query in Geo880 is 6.76 and 6.20 tokens, respectively, and the questions in Geo880 are written using 159 unique natural language tokens. The other is Geo250, a subset of Geo880. Geo250 has only 250 pairs of natural language and formal queries. The average length of the question and the formal query in the Geo250 are 7.48 and 6.47 tokens, respectively, and the questions consist of 270 unique tokens.

The proposed method is evaluated using 10-fold cross validation. The performance of the proposed method is measured in terms of precision and recall. Precision is the percentage of correct questions among all the answered questions, while recall is the percentage of correct questions among all the answerable questions. All the questions of the GeoQuery datasets are answerable. That is, the number of answerable questions is 880 and 250 for Geo880 and Geo250, respectively. The overall performance is given by *F*1*-measure*, the harmonic mean of precision and recall.

### 6.2. Experimental Setup

The proposed method is composed of the generation and the translation stages, as explained in Section 3. Both stages affect the discordance problem, and hence the effectiveness of each stage is analyzed independently. In order to analyze the generation stage, the proposed method is compared with a traditional nearest neighbor method. The nearest neighbor method can be interpreted as a method without a generation strategy because it answers only the questions known in the training dataset. In the generation stage of the proposed method, schema subgraphs are first generated by using Algorithm 1. That is, at least one normalized expression is generated automatically for each schema subgraph from the geography ontology. Further, certain additional expressions for schema subgraphs are collected manually using* PatternGenerator*. For the Geo880 dataset, a human annotator collected an average of 11 normalized expressions per predicate for approximately 2 hours.

The performance of the translation stage relies only on the similarity measurement Sim(*p*, *q*) in Algorithm 2, after normalized expressions are prepared and maintained constant. Two similarity measures, Sim_NI_(*p*, *q*) in (6) and Sim_EU_(*p*, *q*) in (7), have been proposed in this study. The base function of the similarities is a weighted string kernel with the parameters, decay factor and maximum length of a subsequence. The decay factor is set to 0.24 and 0.76 for for semantic codes and lexical terms, respectively, and the maximum length of a subsequence is set to five.

The two similarities are compared with an alignment-based method introduced in our earlier study [28]. When *p* = (*p*_1_,…, *p*_*n*_) is a prepared expression and *q* is a possible expression of a user question, the alignment-based method is defined as

(8) SimALp,q=1n∑i=1nAlignScorepi,qmax⁡AlignScorepi,pi,AlignScoreq,q,

where AlignScore(*p*_*i*_, *q*) returns a matching score between *p*_*i*_ and *q* by a standard alignment method [29]. AlignScore(*p*_*i*_, *q*) has three parameters: (1) the score when two tokens from *p*_*i*_ and *q* match exactly, (2) the score when two tokens do not match, and (3) the score when the matching of two tokens is skipped. The values of the parameters “match,” “mismatch,” and “skip” are set to 1, −3, and −1, respectively.

In Algorithm 2, even the best pair (*p*, *q*) is rejected if their similarity is less than the predefined threshold *θ*. We set different values of *θ* according to the similarity and number of predicates in a prepared expression.  Table 1 shows various values of  *θ* for each similarity. The values of all the parameters, including *θ*, are determined from a pilot experiment with an independent held-out dataset.

### 6.3. Experimental Results

Table 2 presents the evaluation results for the generation stage applied to the Geo880 and Geo250 datasets. In this table, a simplified version of the proposed generation process, Sim_EU−*t*_, is compared with a traditional nearest neighbor with no generation strategy, NN. The simplified version, Sim_EU−*t*_ is the generation part of the proposed method with similarity Sim_EU_ without a threshold strategy, and its translation stage is identical to that of NN. In the absence of a tie, the translation stage always returns a formal query corresponding to the most similar prepared expression. If a tie occurs, the question is rejected and the failure alarm is raised. As shown in Table 2, Sim_EU−*t*_ outperforms NN. NN inevitably suffers from the discordance problem inevitably owing to the existence of unseen formal queries in the test dataset. The result implies that the generation stage of the proposed method contributes significantly to the reduction of the discordance problem.

Table 3 shows the evaluation results for the translation stage applied to the Geo880 and Geo250 datasets. In the table, a comparison of various similarities Sim_NI_, Sim_EU_, and Sim_AL_ is presented. The generation part is fixed and identical prepared normalization expressions are used for the three similarities. From the table, it can be observed that Sim_NI_ achieves the highest performance for Geo250, while Sim_EU_ outperforms the other similarities for Geo880. Sim_EU_ shows stable performances for Geo880 and Geo250, and hence it is considered to be the best similarity. Further, Sim_NI_ and Sim_EU_ always outperform Sim_AL_ for Geo880 and Geo250. Sim_NI_ and Sim_EU_ are based on the expression kernel given in (5), while Sim_AL_ is not. Therefore, it can be inferred that the proposed similarities based on the expression kernel are suitable for measuring similarity between natural language expressions.

These results prove that the proposed method performs well for questions answerable by the geography ontology. However, it cannot be determined whether the proposed method solves the discordance problem because the above experiments do not include unanswerable questions. The GeoQuery dataset consists of only answerable questions. In order to investigate the effectiveness of the proposed method for unanswerable questions, another experiment is performed using a subset of the geography ontology. The subset is created by removing the concept* River* and its related predicates from the geography ontology. Thus, certain questions about a river in Geo880 and Geo250 cannot be answered by this subset. The Geo250 and Geo880 datasets contain 23.6% and 26.5% of river-related questions, respectively.

The results of the experiment are presented in Table 4. When compared with Table 3, Table 4 shows

(a) higher recall values in all cases but lower precision values in most cases;
(b) slightly lower values of *F*1-measures for Sim_NI_ and Sim_EU_ but higher values of *F*1-measure for Sim_AL_.

However, the proposed similarities (Sim_NI_ and Sim_EU_) outperform Sim_AL_, thus implying that the proposed method handles unanswerable questions well.

**Figure 7:**
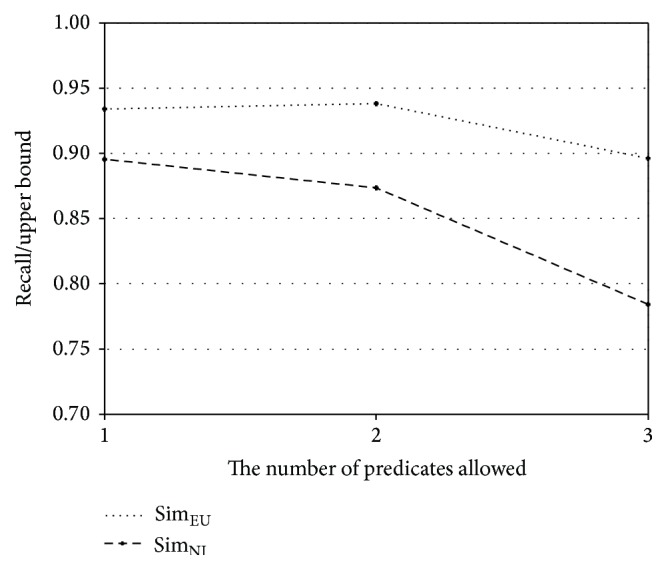
Ratio of recall to upper bound for the original geography ontology.

**Figure 8:**
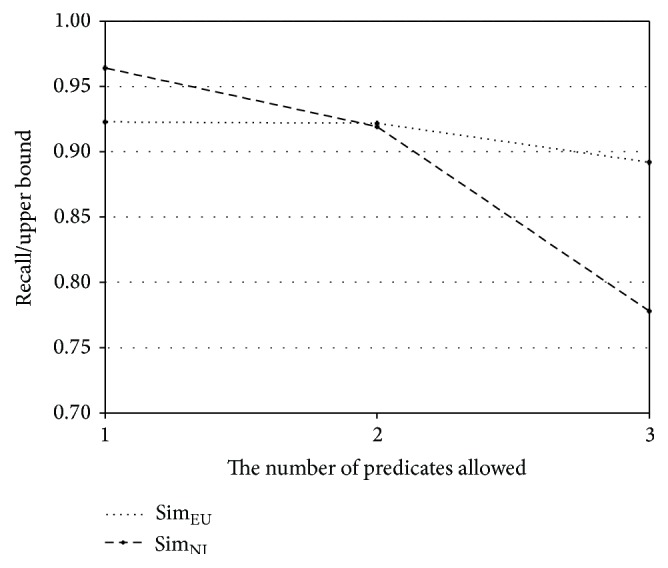
Ratio of recall to upper bound for the subset of the geography ontology.

The performance of the proposed method relies on the number of predicates in a schema subgraph. Thus, recall and precision of Sim_NI_ and Sim_EU_ are further analyzed according to the number of predicates allowed. The experiments for this investigation are performed with Geo880.  Figure 7 shows the ratios of recall to upper bound for the number of allowed predicates in the original geography ontology, and Figure 8 shows the corresponding ratio for the subset of the geography ontology. The upper bound of a recall value is defined as the percentage of the questions that can be expressed as a schema graph among all answerable questions. The upper bounds for the original geography ontology are 38.8%, 77.2%, and 92.8% for one predicate, two predicates, and one predicate and 77.2% for two predicates and three predicates, respectively. The upper bounds for the subset of the ontology are 41.1%, 79.4%, and 94.7% for one predicate, two predicates, and three predicates, respectively. In Figures 7 and 8, the *x*-axis represents the number of predicates allowed, while the *y*-axis represents the ratio of recall to the upper bound. From these figures, it is observed that Sim_EU_ shows a smaller variation than Sim_NI_ as the number of predicates changes. Therefore, Sim_EU_ handles answerable questions more robustly than Sim_NI_, when the normalized expressions corresponding to schema subgraphs are prepared.

**Figure 9:**
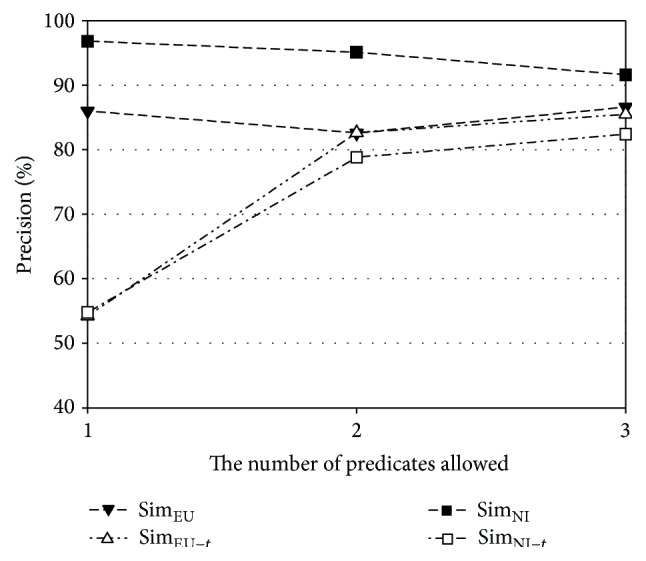
Precision according to the number of predicates for the original geography ontology.

**Figure 10:**
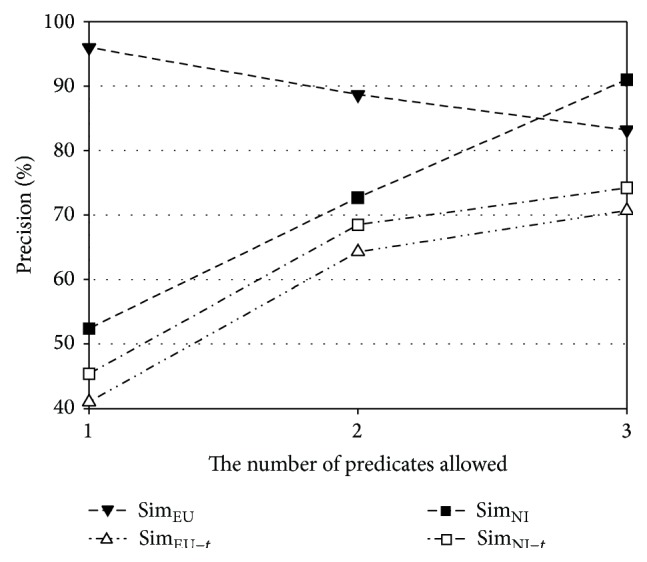
Precision according to the number of predicates for the subset of the geography ontology.

Figure 9 presents precision values with and without the threshold strategy for the original geography ontology, and Figure 10 presents the corresponding values for the subset of the geography ontology. In each graph, the *x*-axis is the number of predicates allowed, while the *y*-axis represents precision. The similarity without the threshold strategy is denoted by attaching “−*t*” to the corresponding label. All similarities with the threshold strategy (Sim_NI_ and Sim_EU_) are always higher than those without the threshold strategy (Sim_NI−*t*_ and Sim_EU−*t*_). Sim_NI_ has the best precision with values always higher than 90%. The improvement in precision with the threshold strategy is also clearly observed in the subset of the geography ontology, thus implying that the use of this strategy effectively blocks unanswerable questions.

### 6.4. Comparison with Other Methods

Sim_EU_ is selected as the similarity to be used because it shows robust results in the experiments above when compared with Sim_AL_ and Sim_NI_. An NLI system is built with the proposed method using Sim_EU_ and the NLI system is compared with WASP [13], Lu08 [30], SEMRESP [15], UBL [31], and UBL-s [31]. WASP is a system that learns transformation rules using statistical machine translation techniques, and Lu08 is an NLI system that uses a generative semantic parser. UBL uses a probabilistic combinatory categorial grammar- (CCG-) based semantic parser, while UBL-s is a UBL that has the ability to skip words. SEMRESP is also based on a semantic parser.

These earlier systems can be divided into two types according to their training data: (1) systems that uses question-formal query pairs as a training dataset: all systems except SEMRESP belong to this type; (2) systems that uses question-answer pairs as a training dataset: SEMRESP is the only system that belongs to this type. The proposed system is different from the above two types. It uses a different type of dataset. In the proposed system, a natural language expression corresponds to a concept or predicate that is used as a formal query component.

The cost of building a training dataset varies according to the dataset type. This cost depends on the labor of human annotators. In the first type of system, human annotators must understand a formal query language completely because in this case, formal queries are used as a part of training data. However, the proposed system requires an understanding of predicates, not the understanding of a language. The second type of system uses question-answer pairs as training data. Collecting answers is simpler than writing formal queries. Therefore, the cost of the proposed system is lower than that of the first type of system, but higher than that of the second type.

In Table 5, the proposed system, denoted as Sim_EU_, is compared with legacy systems. From this table, it is observed that the proposed system has the highest *F*1-measure. The recall value of the proposed system is much higher than that of all other systems; however, its precision is lower than that of Lu08 and WASP. The proposed system shows the best overall performance, thus demonstrating that it is suitable for use as NLI without the need for sophisticated natural language processing.

A concept or a predicate in the proposed system can be interpreted as a simple formal query. Thus, the normalized expressions from the proposed system can be useful for systems of the first type. In order to show this usefulness, UBL is modified to use the normalized expressions obtained from the generation stage of the proposed system. Since UBL is second in terms of performance in Table 5, hence, it is selected for comparison with the proposed system. In Table 6, the proposed system is compared with UBL and a modification of UBL, denoted by UBL-m, applied to the Geo250 dataset. The proposed system, Sim_EU_, outperforms UBL and UBL-m. From this table, a significant observation is that the performance of UBL-m is considerably better than that of UBL. This result proves that it is beneficial to use the proposed generation process in other methods.

**Figure 11:**
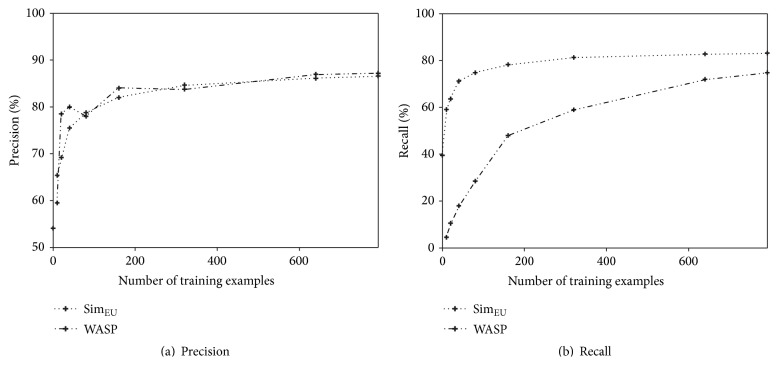
Performance comparison of all systems according to the number of training examples.

The performance of the proposed system depends on the size of training data. In order to observe this dependency, the precision and recall of the proposed system are compared with those of WASP according to the number of training examples, WASP is the only system for which the precision and recall are publicly available, and, therefore, it is selected for comparison. Figures 11(a) and 11(b) depict the values of precision and recall, respectively, for the Geo880 dataset. The precision of the proposed system is similar to that of WASP for all numbers of training examples. However, the recall of the proposed system increases considerably faster than that of WASP. The proposed system with only 40 examples shows recall values similar to WASP that uses all 792 examples. In addition, the proposed system achieves stability with only 320 examples; however, at this point, the recall value is significantly higher than that of WASP with 792 examples.

The proposed NLI system using Sim_EU_ is quantitatively compared to the earlier systems and the results are summarized in three points. First, the potential of our approach without the need for sophisticated natural language processing was verified through the comparison experiments. Note that the proposed NLI system is the only one with no sophisticated natural language processing. As a result, we showed that the proposed method outperforms others in *F*1-measure. Second, the usefulness of normalized expressions from the proposed system was verified in terms of applying the expressions to other systems. For this purpose, an earlier system is compared to its alternative version which utilizes the normalized expressions. The results show that the alternative version outperforms its original system. Lastly, the performance of the proposed NLI system is investigated according to the number of training examples. An efficient system is believed to reach a better performance more quickly with less number of training examples. In this aspect, we showed that the proposed NLI system outperforms an earlier system.

### 6.5. Limitations and Future Work

The proposed system shows high performance; however, it has certain limitations. The limitations of the proposed system are as follows:

- *Linguistic suitability*: in the generation stage of the proposed system, the normalized expressions for a predicate are obtained from the label or identifier of the predicate. However, if the label or identifier is not a legal natural language string, valid expressions cannot be generated. This occurs when the label is absent and the identifier is an abbreviation of two or more words. For example, locIn is often used as an identifier of the predicate “be located in.” However, it cannot be processed accurately, because the proposed system splits it into the two words “loc” and “In.”
- *Scalability*: the number of schema subgraphs with multiple edges increases exponentially with an increase in the number of edges. In order to prevent such a large increase in the number of schema subgraphs, we propose three constraints in Section 4.1.  Table 7 shows the reduction in the number of schema subgraphs for each constraint. Approximately 50% of the schema subgraphs are eliminated by applying constraint 1 for inverse predicates. Constraints 2 and 3 also contribute to the reduction of redundant subgraphs; however, their effect is minor. The performance of the proposed system is not affected by the constraints. Thus, the constraints contribute in increasing the speed of execution of the proposed system without resulting in loss of performance. However, the number of schema graphs remains large even when all constraints are applied.
- *Errors caused by unknown expressions*: the proposed method does not take synonyms into consideration. Hence, the expressions that are synonyms of an existing concept or predicate are considered as unknown expressions. Such expressions result in an error in the proposed system.

The proposed method can be further improved in several aspects. In the generation stage, normalized expressions corresponding to predicates can be enriched by external resources such as the Web. Any sentence with a pair of entities linked by a predicate in a knowledge base is highly likely to express the predicate in a certain way [32], and such sentences can be discovered from the Web. Thus, normalized expressions can be enriched by verifying the expressions from the Web instead of generating new expressions for each predicate. In the translation stage, it will be more useful to incorporate a thesaurus to measure the similarity between normalized expressions. The combination of different similarity measurements is also helpful to compensate the defect of a single similarity measure. We will also extend our work to a large scale knowledge base such as DBPedia [33]. For this purpose, both generation and translation stages will be parallelized with a programming model like MapReduce [34].

We also believe that the proposed method for an NLI can be applied to many other applications. For instance, the generation stage can be used at ontology verbalization which represents the triples of an ontology in a natural language [35]. A normalized expression obtained from the generation stage becomes a candidate expression to verbalize a triple. The expression kernel in the translation stage can be utilized in a nearest neighbor classification which has been studied in various natural language processing tasks such as word sense disambiguation [36], part-of-speech tagging [37], syntactic parsing [38], and machine translation [29]. The existing methods classify a target instance by finding a single known instance matched with the target instance whereas the expression kernel allows a target instance to be matched with a bag of instances belonging to the same class.

## 7. Conclusion

The discordance between the questions interpretable by the interface and those answerable by the knowledge base is a major problem in the field of NLIs. This study proposes a novel method to address this problem. The novelty is summarized in two points. First, in the proposed method, schema subgraphs define all formal queries answerable by the knowledge base explicitly prior to receiving user questions and at least one normalized expression for each schema subgraph is generated in advance. Hence, the proposed method guarantees its users to access all possible formal queries via the prepared expressions. Second, even an unexpected user question is translated into an appropriate formal query by the expression kernel newly proposed in this paper. The expression kernel enables the user question to be matched with its most similar normalized expression. If this matching is confident enough, a final formal query is generated from the schema subgraph corresponding to the matched normalized expression, and otherwise the user question is rejected.

We have shown the effectiveness of the proposed method in reducing the discordance problem through two steps of experiments. In the first step, it has been shown that the proposed system achieves high performance for answerable questions and rejects unanswerable questions effectively. In the second step, the comparison of the proposed method with five legacy systems demonstrates that the proposed system outperforms all the legacy systems. The usefulness of normalized expressions obtained from the generation stage also has been shown by applying the expressions to a legacy system, UBL. These experimental results prove that the proposed method is suitable as a practical NLI without sophisticated natural language processing techniques.

## Figures and Tables

**Algorithm 1:**
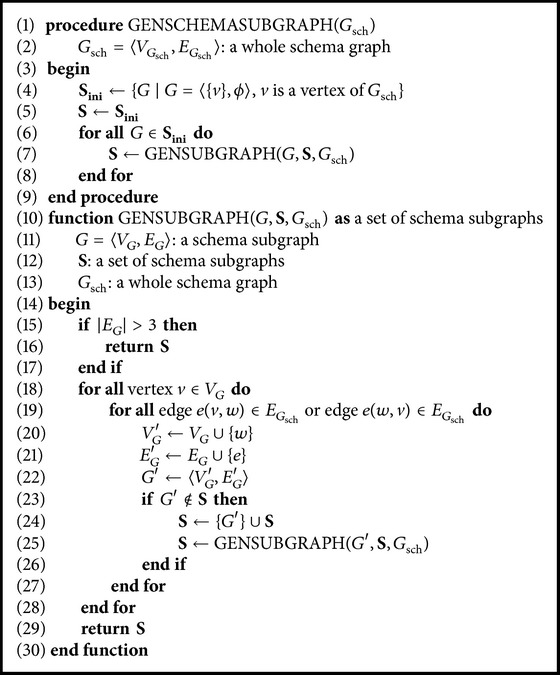
The schema subgraph generation algorithm.

**Algorithm 2:**
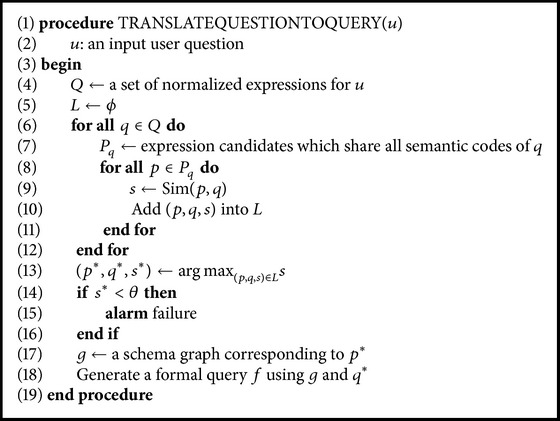
The user question translation algorithm.

**Table 1:** Thresholds for three similarities according to the number of predicates.

Similarity	Number of predicates			
	0	1	2	3
Sim_AL_	1.00	0.41	0.35	0.28
Sim_NI_	1.00	0.72	0.72	0.81
Sim_EU_	1.00	0.36	0.27	0.25

**Table 2:** Results from the evaluation of the generation stage using the geography ontology.

Dataset	Similarity	Precision	Recall	*F*1-measure
Geo880	NN	66.1	36.7	47.1
	Sim_EU−*t*_	**85.5**	**83.3**	**84.4**

Geo250	NN	76.0	52.8	62.0
	Sim_EU−*t*_	**90.1**	**88.7**	**89.4**

**Table 3:** Results from the evaluation of the translation stage using the geography ontology.

Dataset	Similarity	Precision	Recall	*F*1-measure
Geo880	Sim_AL_	77.4	70.5	73.8
	Sim_NI_	**91.6**	72.8	81.1
	Sim_EU_	86.6	**83.2**	**84.9**

Geo250	Sim_AL_	90.7	85.0	87.7
	Sim_NI_	**96.3**	**86.6**	**91.1**
	Sim_EU_	90.6	**86.6**	88.5

**Table 4:** Results from the evaluation of the generation stage using a subset of the geography ontology.

Dataset	Similarity	Precision	Recall	*F*1-measure
Geo880	Sim_AL_	79.2	72.4	75.6
	Sim_NI_	**91.0**	73.7	81.4
	Sim_EU_	83.2	**84.5**	**83.8**

Geo250	Sim_AL_	92.8	87.0	89.7
	Sim_NI_	**94.7**	87.2	**90.7**
	Sim_EU_	88.8	**87.3**	88.0

**Table 5:** Performance comparison of the proposed system with legacy systems on the Geo250 dataset.

Systems	Precision	Recall	*F*1-measure
WASP	**95.4**	70.0	80.8
Lu08	91.5	72.8	81.1
UBL	88.2	78.1	82.7
UBL-s	80.8	80.4	80.6
SEMRESP	—	73.2	—
Sim_EU_	90.6	**86.6**	**88.5**

**Table 6:** Performance comparison of the proposed system with UBL and UBL modification on the Geo250 dataset.

Method	Precision	Recall	*F*1-measure
UBL	88.2	78.1	82.7
UBL-m	**93.5**	81.6	**87.1**
Sim_EU_	90.6	**86.6**	**88.5**

**Table 7:** The effect of the constraints on the number of generated schema subgraphs.

Constraint	Number of predicates allowed		
	1	2	3
No constraint	182	11,627	232,367
Constraint 1 (inverse relationship)	151	8,288	136,443
Constraint 2 (functional)	182	11,623	232,293
Constraint 3 (inverse functional)	182	11,626	232,313
All constraints applied	151	8,283	136,391
